# Macrophage-derived extracellular vesicles mediate smooth muscle hyperplasia: role of altered miRNA cargo in response to HIV infection and substance abuse

**DOI:** 10.1096/fj.201701558R

**Published:** 2018-04-19

**Authors:** Himanshu Sharma, Mahendran Chinnappan, Stuti Agarwal, Pranjali Dalvi, Sumedha Gunewardena, Amy O’Brien-Ladner, Navneet K. Dhillon

**Affiliations:** * Division of Pulmonary and Critical Care Medicine, Department of Medicine, University of Kansas Medical Center, Kansas City, Kansas, USA; and; † Department of Molecular and Integrative Physiology, University of Kansas Medical Center, Kansas City, Kansas, USA

**Keywords:** HIV-PAH, pulmonary vascular remodelling, miR-130a, cocaine

## Abstract

Our previous studies consistently demonstrate enhanced pulmonary vascular remodeling in HIV–infected intravenous drug users, and in simian immunodeficiency virus–infected macaques or HIV-transgenic rats exposed to opioids or cocaine. Although we reported an associated increase in perivascular inflammation, the exact role of inflammatory cells in the development of pulmonary vascular remodeling remains unknown. In this study, HIV–infected and cocaine (H+C)–treated human monocyte derived macrophages released a higher number of extracellular vesicles (EVs), compared to HIV-infected or uninfected cocaine-treated macrophages, with a significant increase in the particle size range to 100–150 nm. Treatment of primary human pulmonary arterial smooth muscle cells (HPASMCs) with these EVs resulted in a significant increase in smooth muscle proliferation. We also observed a significant increase in the miRNA-130a level in the EVs derived from H+C-treated macrophages that corresponded with the decrease in the expression of phosphatase and tensin homolog and tuberous sclerosis 1 and 2 and activation of PI3K/protein kinase B signaling in HPASMCs on addition of these EVs. Transfection of HPASMCs with antagomir-130a–ameliorated the EV-induced effect. Thus, we conclude that EVs derived from H+C-treated macrophages promote pulmonary smooth muscle proliferation by delivery of its prosurvival miRNA cargo, which may play a crucial role in the development of PAH.—Sharma, H., Chinnappan, M., Agarwal, S., Dalvi, P., Gunewardena, S., O’Brien-Ladner, A., Dhillon, N. K. Macrophage-derived extracellular vesicles mediate smooth muscle hyperplasia: role of altered miRNA cargo in response to HIV infection and substance abuse.

The risk of developing pulmonary arterial hypertension (PAH) in HIV–infected individuals is 6- to 8-fold more than in individuals without HIV infection (1), with prevalence ranging between 0.4 and 0.6% (2), in comparison to prevalence of only 0.05% idiopathic PAH (3). However, recent reports claim the prevalence of PAH ranging from 2.6 to 15.5% in HIV-infected patients (3–7). In the past decade, the use of antiretroviral therapy gained importance in effectively suppressing HIV replication and increasing the life expectancy of HIV-infected individuals. However, the reports regarding the effect of antiretroviral therapy in causing or preventing HIV-PAH are still contradictory, and mortality is high among patients with HIV-PAH (1, 8), thereby making HIV-PAH one of the most critical noninfectious complications related to HIV infection. Recent studies and meta-analyses have identified intravenous drug use (IVDU) to be the greatest risk factor for the development of pulmonary hypertension among HIV-infected individuals, accounting for almost 50–80% of the HIV-infected population. Our laboratory has broadly investigated the potential link between HIV-associated PAH and IVDU and has shown that a dual hit of drugs of abuse, such as opioids and cocaine, in HIV/simian immunovirus (SIV)–infected humans/macaques and HIV-transgenic (Tg) rats augments the pulmonary vascular remodelling associated with development of PAH (9–12).

The histopathological changes in HIV-PAH including angio-obliteration and plexiform lesions involve abnormal proliferation of pulmonary vascular smooth muscle cells (SMCs) and endothelial cells (13). This finding is interesting because no evidence has been found of viral DNA/RNA in pulmonary vessels (14, 15). However, studies demonstrate direct action of HIV proteins released by the infected lymphocytes and macrophages in the associated vascular dysfunction (16). We previously reported direct effect of HIV proteins such as transactivator of transcription (Tat) in promoting smooth muscle proliferation that synergizes in the presence of cocaine, opioids, or methamphetamine (9–12, 17) by activation of various angiogenic and cell proliferation pathways.

Inflammation is one of the key players in the prognosis of the vascular remodelling associated with PAH. We observed significant perivascular inflammation and infiltration of macrophages around the remodelled vessels in the lung sections from HIV-Tg rats treated with cocaine, as well as in the lung sections from HIV-infected opioid and cocaine users (11, 18). Apart from contributing to pulmonary vascular remodeling by direct release of viral proteins, growth factors, and cytokines, inflammatory cells may communicate with vascular cells through release of extracellular vesicles (EVs). EVs are membrane vesicles released naturally by most cell types and, depending on size, are classified into various types including exosomes (∼40–150 nm) and microvesicles (150–1000 nm). These EVs act as mediators of cell–cell communication by transferring its contents, such as proteins (*e.g*., growth factors, cytokines, and transcription factors) and nucleic acids (RNAs and miRNAs), to neighboring cells when fused with the target cell membrane (19). Several studies reported association of changes in the EV cargo with the pathologic processes of various diseases, including PAH (20–23). Therefore, it became interesting to investigate the role of EVs derived from HIV-infected and drug of abuse–treated inflammatory cells in the pathogenesis of PAH.

In the current study, we report the alterations in the number and cargo of EVs released by monocyte-derived macrophages (MDMs) on HIV infection in the presence or absence of cocaine and the involvement of these EVs in augmenting the proliferation of pulmonary arterial SMCs. We demonstrate that alterations in the expression of molecules involved in the prosurvival PI3K/protein kinase B (AKT) signaling cascade in SMCs on receiving the miRNA cargo from EVs lead to smooth muscle hyperplasia.

## MATERIALS AND METHODS

### Cell culture and treatments

Monomac-1, a human monocytic cell line, was grown in suspension in Rosewell Park Memorial Institute (RPMI)-1640 medium supplemented with 10% fetal bovine serum (FBS) (Atlanta Biologicals, Flowery Branch, GA, USA), 2 mM glutamine, sodium pyruvate, and penicillin-streptomycin and differentiated with 20 ng/ml phorbol 12-myristate 13-acetate (MilliporeSigma, Burlington, MA USA) for 72 h. In addition, human primary MDMs were isolated from healthy human peripheral blood mononuclear cells and were cultured in RPMI-1640 supplemented with 20 μg/ml granulocyte–macrophage colony-stimulating factor and 5 μg/ml monocyte colony-stimulating factor for 7 d (24). Differentiated MDMs were pretreated with 1 µM cocaine hydrochloride (MilliporeSigma) followed by infection with 5 ng/ml HIV_Bal_ (25–27). After infection, the cells were grown for 2 and 4 d in the presence or absence of cocaine (1 µM) (9, 11, 24, 28) in exosome-free culture medium (RPMI containing serum depleted of EV contaminants by overnight centrifugation at 100,000 *g*). The cell culture supernatants were collected at d 2 and 4 for EV isolation.

Primary human pulmonary arterial smooth muscle cells (HPASMCs) (ScienCell Research Laboratories, Carlsbad, CA, USA) were grown in SMC medium (ScienCell Research Laboratories) supplemented with 2% FBS, SMC growth supplements, and penicillin-streptomycin. At 80% confluence, the cells were serum starved for 48 h followed by treatment with EVs isolated from MDMs treated with H+C or with cocaine treatment alone.

### Isolation and characterization of EVs

For isolation of EVs, the cell culture supernatants were collected and centrifuged at 2000 rpm for 10 min, followed by filtration through 0.22 µm filter to remove all cell debris and particles. The supernatants were then centrifuged twice at 100,000 *g* for 70 min with a PBS wash in between. The EVs were then suspended in PBS and stored at −80°C until they were used. The size distribution and number of EVs obtained was analyzed with the Nanosight LM10 system (Malvern Panalytical, Malvern United Kingdom) by applying a monochromatic 404 nm laser to 500 μl diluted EVs and measuring the Brownian movement of each particle. Video files of 60 s duration at a rate of 25 frames/s were recorded and analyzed in the Nanoparticle Tracking Analysis software (Malvern Panalytical). The mean, mode, and median vesicle size and the number of exosomes in each sample were thus obtained and corrected by the dilution factor before final quantification. The ratio of the EVs and cellular protein was assessed by bicinchoninic acid assay. Transmission electron microscopy (TEM) was performed after negative staining of EV preparation using uranyl acetate (29). Further, EVs were also characterized by Western blot analysis with antibodies for the specific exosome markers cluster of differentiation (CD)-9, asparagine-linked glycosylation-2 interacting protein X, acetylcholinesterase, and tumor-susceptibility gene 101, as well as negative markers, such as heat shock protein 60 and lysosomal-associated membrane protein-1. The viral load in HIV-infected cell supernatants and EVs was measured with an ELISA for viral glycosaminoglycan p24 (Beckman Coulter, Brea, CA, USA). Total RNA quality extracted from EVs was tested on a Bioanalyzer from Agilent Technologies (Santa Clara, CA, USA).

### Cell proliferation assay

HPASMCs (3 × 10^3^ cells/well) were seeded in 96-well plates for 72 h. After 24 h of starvation in 0.1% FBS SMC medium, the cells were treated with EVs (2 µg/well) or supernatants from H+C or cocaine-treated MDMs in the presence or absence of the exosome inhibitor GW4869 for 48 or 96 h. Proliferation of HPASMCs was assessed by using CellTiter 96 Aqueous One Solution Cell Proliferation Assay (MTS; Promega, Madison, WI, USA), according to the manufacturer’s protocol.

### Transfection

Primary HPASMCs were transiently transfected with chemically modified single-strand mirVana miRNA antagomirs of either miR-130a or scrambled control (Thermo Fisher Scientific, Waltham, MA, USA) using HiPerfect reverse transfection reagent (301704; Qiagen, Germantown, MD, USA), according to the manufacturer’s instructions. After transfection, cells were starved for 24 h with 0.1% serum containing SMC medium followed by EV treatment and mRNA or proliferation analysis.

### RNA isolation and real-time PCR analysis

For RNA isolation from EVs, 32 µg EVs were spiked with 1 µl (5 nM) Cel-miR-39-3p miRNA (30) of *Caenorhabditis elegans*, which lacks homology with human miRNA (MSY0000010; Qiagen) before RNA isolation. Exosomal RNA was isolated by using the miRNeasy Kit (Qiagen), and cDNAs specific to miRNAs were prepared with the miScript II RT Kit (Qiagen), according to the manufacturer’s instructions. Total RNA isolation from HPASMC, treated with EVs or left untreated was performed with Trizol reagent (Thermo Fisher Scientific) followed by the cDNA synthesis for mRNAs by using the iScript cDNA Synthesis Kit (Bio-Rad, Hercules, CA, USA), per the manufacturer’s protocol. The mRNA or miRNA expression was analyzed by standard quantitative RT-PCR protocol, as reported by Dalvi *et al*. (11).

### Small-RNA sequencing and pathway analysis

The Illumina HiSeq2500 Sequencing System at the University of Kansas Medical Center Genomics Core was used for small-RNA sequencing (small-RNA seq). Extracellular vesicle RNA was used to initiate the TruSeq Small RNA library preparation protocol (RS200-0012 Kit A, RS200-0024 Kit B, RS200-0036 Kit C, and RS200-0048 kit D; Illumina, San Diego, CA, USA). The global unbiased miRNA profiles of all samples were interrogated by using Illumina’s small-RNA seq technology (TruSeq Small RNA Sample Prep Kit). The small-RNA seq samples were sequenced for 50-cycle single end reads using the HiSeq2500 Sequencing System. The small-RNA seq reads with noncanonical letters (*e.g.*, N) were first removed from the samples. Adaptors were clipped from the remaining reads. The resulting reads that were shorter than 17 base pairs were discarded. The remaining reads were mapped to the human genome (GRCh38.rel77) by using Bowtie (31). The read abundance estimates of all known human miRNAs (mirBase v.21) were computed with miRDeep2 (32).

The miRNA expression counts obtained from this process were analyzed with a negative binomial generalized linear model from the edgeR package (33), to identify statistically significant, differentially expressed miRNAs between the control and the 3 treatment conditions: HIV, cocaine, and H+C. The significant *P*-values were adjusted for multiple hypotheses testing by the Benjamini and Hochberg method (34).

Pathway analysis for statistically significant and differentially expressed miRNAs in H+C EVs *vs.* control was performed with the Diana mirPath v.3 tool (35), which combines the information of miRNA targets that are predicted using microT-CDS v.5 algorithm with the corresponding Kyoto Encyclopedia of Genes and Genomes pathway. Differentially expressed miRNAs targeting PI3-Akt signaling were analyzed, and the network was generated through the use of Ingenuity Pathway Analysis (IPA; Qiagen).

### Western blot analysis

Primary HPASMCs treated with or without EVs were lysed with RIPA lysis buffer (Cell Signaling Technology, Danvers, MA, USA) and then used for Western blot analysis as reported by Dhillon *et al*. (9). Western blots were probed with antibodies against AKT, p-AKT, p70S6K, phospho-p70S6K, glycogen synthase kinase-3αβ, p-glycogen synthase kinase-3αβ, c- poly ADP ribose polymerase, phosphatase and tensin homolog (PTEN), tuberous sclerosis-1 and -2, and proliferating cell nuclear antigen (PCNA; Cell Signaling Technology). The horseradish peroxidase–conjugated anti-mouse or -rabbit secondary antibodies (MilliporeSigma) were used with the ECL system for detection (Thermo Fisher Scientific). NIH ImageJ software (National Institutes of Health, Bethesda, MD, USA) was used for densitometric analysis of immunoblots.

### Statistical analysis

Statistical analysis was performed by 1-way ANOVA with a *post hoc* Bonferroni correction for multiple comparisons. Two-sided *P*-values were calculated for analyzing all *in vitro* experiments (Prism; GraphPad, La Jolla, CA, USA). The results were judged statistically significant with Bonferroni-corrected values of *P* < 0.05.

## RESULTS

### Increased secretion of EVs by HIV-1–infected macrophages upon cocaine treatment

**Figure 1:**
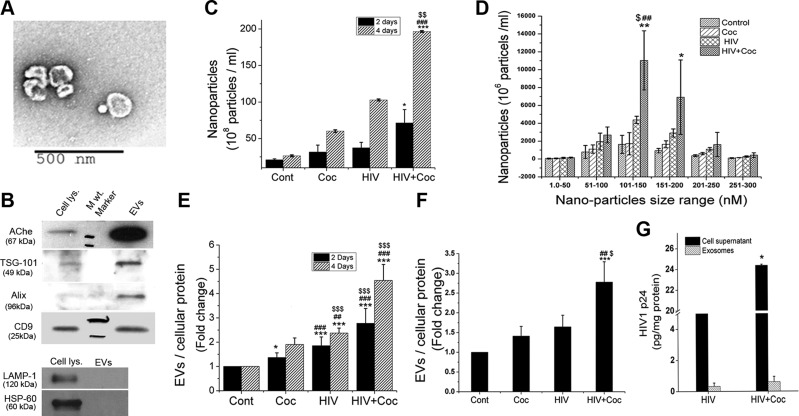
Increased release of EVs on cocaine treatment of HIV-infected MDMs. The human monomac-1 cell line and primary monocytes isolated from human peripheral blood mononuclear cells were differentiated into MDMs followed by HIV-1 bronchoalveolar lavage (5 ng/ml) infection, 1 µM cocaine (Coc) treatment, or both. EVs were isolated from supernatants collected at d 2 and 4 after infection. Isolated exosomes were analyzed for their purity, shape, and size distribution. *A*) Representative TEM micrograph showing EVs. Original magnification, ×10,000. *B*) Western blot showing comparison of exosomal protein expression in cells and isolated EVs. *C*, *D*) Quantification of EVs from monomac-1–derived macrophages. *E*, *F*) Changes in EV:cellular protein ratio after H+C treatment of macrophages derived from monomac-1 cells (*E*) and human primary monocytes (*F*). *G*) HIV-1 p24 levels in cellular supernatants and EV extracts, as measured by ELISA. Data are means ± sd from ≥3 independent experiments. *P < 0.05, **P < 0.01, ***P < 0.001 *vs.* control; ^##^*P* < 0.01, ^###^*P* < 0.001 *vs.* cocaine; ^$^*P* < 0.05, ^$$^*P* < 0.01, ^$$$^*P* < 0.001 *vs.* HIV.

EVs released by HIV-infected monomac-1–derived macrophages, treated with cocaine or untreated were isolated from culture supernatants at 2 and 4 d after infection. TEM was performed to assess the morphology and size of the obtained vesicles, and 40–250 nm, cup-shaped microvesicles were observed by analysis after negative staining of the EV preparation with uranyl acetate (**Fig. 1*A***). To further confirm the presence of EVs (30–150 nm) in the vesicle preparation, protein extracts from EVs and whole-cell lysate were analyzed for exosomal markers by Western blot (Fig. 1*B*). The exosomal markers tumor-susceptibility gene 101, asparagine-linked glycosylation-2 interacting protein X, and cluster of differentiation 9 were highly enriched in EV lysate, whereas lysosomal-associated membrane protein-1 and heat shock protein-60 were observed only in cell lysate. These findings suggested that the EV preparation is highly enriched in exosomes. Quantification of EVs by NanoSight revealed a higher concentration of total nanoparticles in the preparation from H+C-treated monomac-1 cells than that in the other 3 groups, at both 2 and 4 d after infection (Fig. 1*C*). In addition to an increase in the number of particles ranging from 51 to 100 nm, a significant increase in the levels of 101–150 nm particles was observed (Fig. 1*D*). To exclude the possibility that the alterations in the number of EVs released is not related to a corresponding change in number of cells, but is induced by H+C treatment, the ratio of total exosomal protein to cellular protein was calculated. We found significantly higher EV protein concentrations in H+C-of cocaine-treated monomac-1 cells, as well as primary human MDMs, when compared with untreated control (Fig. 1*E, F*). Furthermore, protein concentration of EV preparation from H+C-treated cells was significantly higher than in EVs isolated from only HIV-1-infected or cocaine-treated cells (Fig. 1*E, F*). Although we did not separate HIV-1 virions from exosomal preparation, analysis of HIV capsid protein: p24 by ELISA demonstrated significant increase in the levels of p24 in cellular supernatants from HIV-infected primary human MDMs on exposure to cocaine, as reported by us (24), but relatively very little p24 was observed in the corresponding EV extracts (Fig. 1*G* and Supplemental Fig. S1).

### EVs derived from H+C–treated macrophages promote proliferation of pulmonary arterial SMCs

**Figure 2:**
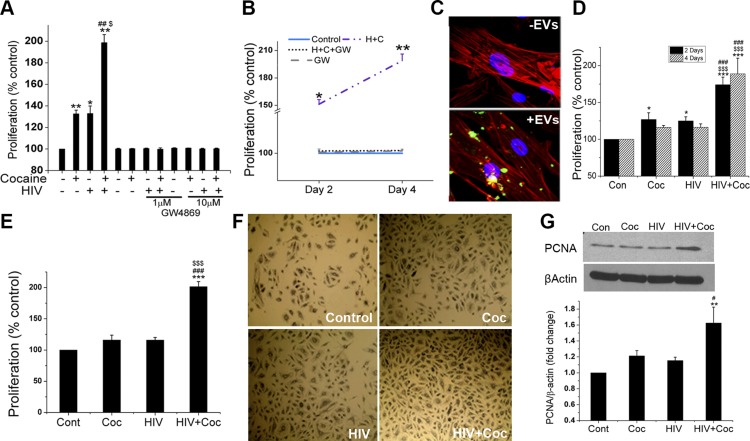
Increased SMC proliferation on uptake of H+C- or cocaine-treated MDM–derived EVs. *A*, *B*) Monomac-1–derived macrophages were treated with H+C in the presence or absence of GW4869 (1 or 10 μM). Supernatants collected were added on HPASMCs in 1:1 ratio with SMC medium followed by a cell proliferation assay after 2 and 4 d. *C*) Uptake of EVs by SMCs. EVs were labeled with fluorescent PKH67 dye (green) and added on HPASMCs (10 µg/24 well) for 2 h. Cells were later washed and fixed, followed by staining with phalloidin (red). *D*, *E*) Exosomes (2 μg) resuspended in PBS, isolated from untreated, cocaine (Coc) treated, HIV-1 infected and from both HIV-1-infected and cocaine-treated monomac-1 (*D*) or primary human monocyte (*E*) derived macrophages were added to 0.1% serum-starved HPASMCs overnight followed by replenishing the medium the next day. MTS cell proliferation assay was performed at 48 h after exosome treatment. *F*) Representative phase-contrast images of HPASMCs after MTS assay. *G*) Western blot analysis of HPASMC total cell extract after 48 h of EV treatment for proliferation marker, PCNA. Data are means ± sd from ≥3 independent experiments. *P < 0.05, **P < 0.01, ***P < 0.001 *vs.* control; ^#^*P* < 0.05, ^##^*P* < 0.01, ^###^*P* < 0.001 *vs.* cocaine; ^$^*P* < 0.05, ^$$$^*P* < 0.001 *vs.* HIV.

We reported enhanced proliferation of pulmonary arterial SMCs in lung sections from HIV-infected humans (11) and SIV-infected macaques (10) or in lungs from noninfectious HIV-transgenic rats in the presence or absence of illicit drugs (12). Given that these cells do not support productive viral replication, we hypothesized that EVs released into the vascular environment by surrounding HIV-infected inflammatory cells may play a crucial role in vascular remodeling. To test this theory, we first blocked the release of exosomes from macrophages by incubating the cells in the presence or absence of the exosome release inhibitor GW4869 during treatment with cocaine, HIV-1 infection, or H+C. Supernatants from these macrophages were then added over primary HPASMCs to assess proliferation. As expected, HPASMCs treated with supernatants from cells exposed to HIV-1, cocaine, or H+C in the absence of GW4869 showed significant increase in cell proliferation, compared to cells exposed to EVs from untreated macrophages (**Fig. 2*A, B***). However, HPASMCs exposed to the conditioned medium from MDMs with the same corresponding treatment in the presence of GW4869 did not result in the increased proliferation. Next, we tested whether EVs are internalized and taken up by primary HPASMCs. PKH67-labeled EVs were added to HPASMCs, and intracellular localization of the vesicles within the cells was assessed by immunofluorescence. Representative confocal images (Fig. 2*C*) clearly show uptake of green fluorescence-labeled EVs inside phalloidin-stained (red fluorescence) SMCs within 2 h of exposure. Consequently, direct exposure of quiescent primary HPASMCs to EVs harvested from H+C-treated monomac-1 cells (Fig. 2*D*) or primary human MDMs (Fig. 2*E*) for 48 h resulted in a synergistic increase in cell proliferation compared to cells treated with EVs derived from macrophages exposed to a single treatment (HIV-1 or cocaine). Corresponding phase-contrast images of the cell culture wells after a 48-h treatment with EVs were obtained (Fig. 2*F*). Furthermore, these findings were also supported by the increased expression of the cell proliferation marker PCNA in the cellular extract from HPASMCs treated with EVs derived from H+C-treated MDMs (Fig. 2*G*). These findings confirm that EVs released from H+C-, HIV, or cocaine-treated MDMs promote SMC proliferation.

### Increased levels of miR-130a in EVs derived from HIV-infected and cocaine-treated macrophages

EVs act as mediators of cell–cell communication by transferring its contents, such as proteins and nucleic acids (mRNA, miRNA) to neighboring cells. We next explored whether the alterations in the miRNA cargo of EVs derived from HIV-infected, cocaine-treated macrophages play any role in modulating smooth muscle proliferation. For a pilot analysis of miRNA cargo within EVs, we performed a small-RNA seq on EV samples from 4 treatment groups (H+C, HIV, cocaine, and control) using 2 sets of human MDMs isolated from 2 biologically different donors. The quality of total RNA extracted from EVs was first tested on a Bioanalyzer, which revealed a substantial amount of small RNA (<30 nt) and a very low amount of rRNA (18S and 28S; data not shown). Small-RNA seq analysis of EVs revealed differential regulation (>1.5-fold change) of 185 miRNAs in the H+C, 173 in the HIV, and 175 in the cocaine group when compared with EVs from untreated, uninfected control MDMs. Although variability would be expected in MDMs from 2 different donors, we found 17 miRNAs significantly up- or down-regulated (*P* < 0.05) in EVs from the H+C MDM group, whereas only 4 and 5 miRNAs were found in HIV-infected and cocaine-only–treated cells, respectively, when compared with the uninfected control. Moreover, the EVs from H+C group contained 18 miRNAs that were significantly differentially expressed when compared with miRNA cargo of EVs from HIV- and cocaine-treated MDM groups. Furthermore, 41, 36, and 29 differentially regulated miRNAs in the H+C, HIV, and cocaine groups, respectively, were found to be involved in the proliferation of cells as determined by IPA (Qiagen). When pathway analysis of differentially expressed proliferative miRNAs was performed using Diana miRPath (35), we identified the ErbB, TGF-β, mTOR, PI3K/Akt, VEGF, Wnt, MAPK, and Hippo pathway as some of the most significant pathways potentially up-regulated in the H+C group (Supplemental Fig. S2*A*). Given that PI3K/AKT is common to the downstream signaling of ErbB, VEGF, platelet-derived growth factor, and other receptor tyrosine kinases, we next focused on miRNAs involved in PTEN/PI3K/mTOR signaling. The differential expression of miRNAs potentially targeting PI3K/AKT cascade in all 3 groups when compared with the control and in the H+C group when compared with both the HIV alone and cocaine alone groups are represented as a clustergram in Supplemental Fig. S2*B*. Based on the analysis of these differentially expressed PI3K/AKT signaling miRNAs according to IPA software, we selected 3 significantly upregulated miRNAs: miR-10a, -130a, and -27a and 2 significantly downregulated miRNAs, miR-181 and -200, for further analysis.

**Figure 3:**
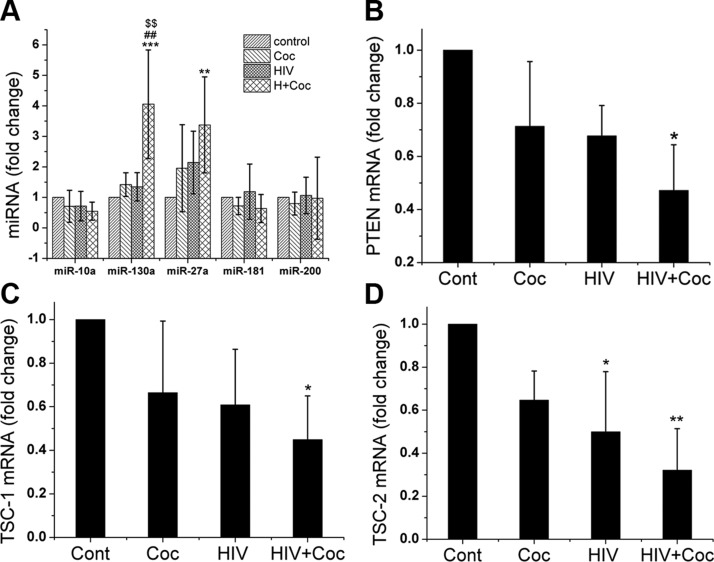
Increased expression of miR-130a and -27a in EVs derived from H+C-treated macrophages. *A*) Quantitative RT-PCR analyses of miR-130a, -130a, -27a -10a, -181, and -200 in EVs derived from MDMs that have been HIV-infected, cocaine-treated (Coc), or both. *B*–*D*) Quiescent HPASMCs were treated with EVs in serum-free SMC medium for 16 h followed by RNA extraction and quantitative RT-PCR analysis of PTEN (*B*) and TSC-1 (*C*) and -2 (*D*) mRNAs. Data are means ± sd from ≥3 independent experiments. **P < 0.01, ***P < 0.001 *vs.* control; ^##^*P* < 0.01 *vs.* cocaine; ^$$^*P* < 0.01 *vs.* HIV.

First, we validated the differential expression of these selected miRNAs by performing quantitative RT-PCR on total RNA extracted from various EVs preparations. A significant increase in the expression level of miR-130a and -27a was observed in EVs from the H+C group when compared with those from the untreated group (**Fig. 3*A***). miR-130a that showed a maximum increase in the H+C group *vs.* untreated control was the only miRNA found to be significantly elevated in H+C EVs when compared with EVs from the HIV- or cocaine-only groups. No significant changes were observed in the levels of miR-10a, -181, or -200 in any of the groups (36).

### Activation of the PI3K/AKT signaling pathway in HPASMCs on exposure to EVs derived from HIV-1–infected and cocaine-treated macrophages

**Figure 4:**
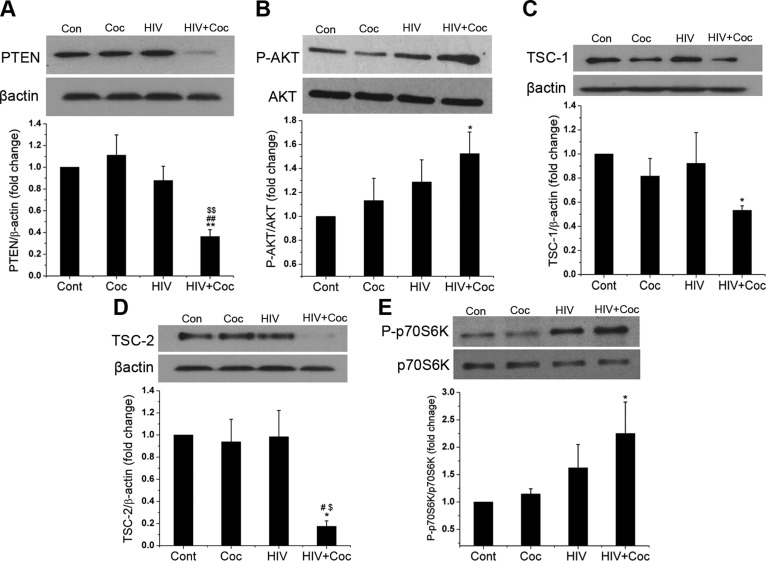
H+C-treated macrophage–derived EVs activate PI3K/AKT signaling in HPASMCs. Quiescent HPASMCs were treated with EVs derived from H+C-, HIV-1-, or cocaine (Coc)-treated MDMs in serum-free SMC medium for 16 h followed by cell lysis with RIPA buffer for Western blot analysis of PTEN (*A*), p-AKT (*B*), TSC-1 (*C*), TSC-2 (*D*) and phospho-p70S6K (*E*). Means ± sem from ≥3 independent experiments. *P < 0.05, **P < 0.01 *vs.* control; ^#^*P* < 0.05, ^##^*P* < 0.01 *vs.* cocaine; ^$^*P* < 0.05, ^$$^*P* < 0.01 *vs.* HIV.

The up-regulated miR-130a has been reported to directly target PTEN, a tumor-suppressor protein that negatively regulates phosphorylation and activation of AKT (37–40). In addition, miR-130a is known to target tumor-suppressor complexes (TSCs)-1 and -2, a cell growth inhibitor negatively regulated by AKT (41). Therefore, we next tested whether the enhanced smooth cell proliferation on exposure to EVs is by targeting PTEN and TSC-1/2 molecules of PI3K/AKT proliferative signaling cascade. We examined both mRNA and protein expression of PTEN after adding EVs derived from H+C-, HIV-1-, or cocaine-treated MDMs to primary HPASMCs for 16 h. Both mRNA and protein levels of PTEN were significantly reduced in HPASMCs treated with H+C EVs, as shown by real-time PCR (Fig. 3*B*) and Western blot (**Fig. 4*A***) analysis when compared with other treatments or untreated control. This result correlated with the significant up-regulation of AKT phosphorylation in HPASMCs treated with H+C EVs (Fig. 4*B*). When we tested the levels of TSC1 and -2 in SMCs treated with EVs from H+C-, HIV-1-, or cocaine-treated MDMs, we observed significant decrease in the mRNA (Fig. 3*C, D*), as well as protein expression (Fig. 4*C, D*) of both TSC1 and -2 in cells treated with H+C EVs when compared with untreated controls. Furthermore, this downregulation of TSC1 and -2 corresponded with the activation of downstream P70S6K as shown by increase in phosphorylated levels of P70S6K in HPASMCs treated with H+C EVs (Fig. 4*E*).

### EV-associated miR-130a promotes smooth muscle proliferation

**Figure 5:**
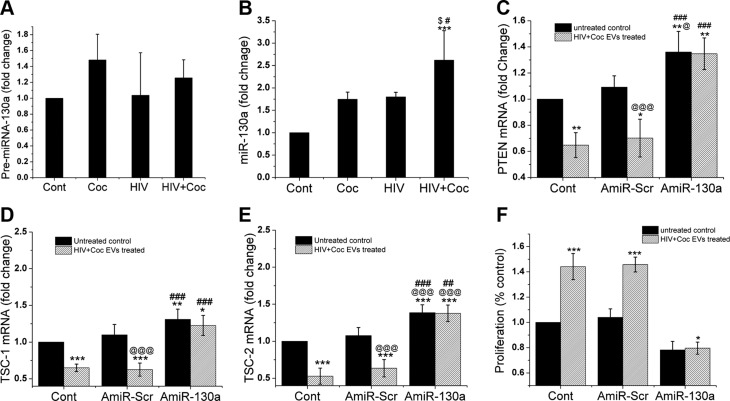
Silencing of miR-130a in SMCs prevents H+C derived EVs mediated inhibition of PTEN expression and hyperproliferation. *A*, *B*) Quiescent HPASMCs were treated with exosomes derived from HIV-infected, cocaine-treated (Coc), or H+C-treated macrophages or those for 16 h followed by RNA extraction, for RT-PCR analysis of precursor (*A*) and mature (*B*) miR-130a expression. *C*–*F*) HPASMC were transfected with antagomirs-130a (AmiR-130a) or scrambled miRNA (AmiR-Scr). At 24 h post-transfection cells were made quiescent for 24 h in 0.1% FBS–containing medium followed by addition of H+C EVs for 16 h to perform PTEN (*C*), TSC-1 (*D*), TSC-2 (*E*) mRNA analysis and for 48 h to perform cell proliferation assay (*F*). Data are means ± sd from ≥3 independent experiments. *P < 0.05, **P < 0.01, ***P < 0.001 *vs.* control; ^@^*P* < 0.05, ^@@@^*P* < 0.001 *vs.* untreated AmiR-Scr; ^#^*P* < 0.05, ^##^*P* < 0.01, ^###^*P* < 0.001 *vs.* exosome treated AmiR-Scr.

After confirming a significant increase in the levels of miR-130a in H+C EVs and downregulation of PTEN and TSC-1/2 expression upon addition of these EVs to HPASMCs, we next investigated whether miR-130a is transferred from EVs and directly involved in the down-modulation of PTEN and TSC-1/2 in the recipient SMCs. First, we examined precursor miR-130a and mature miR-130a levels in HPASMCs after adding EVs derived from H+C-, HIV-1-, or cocaine-treated macrophages (**Fig. 5*A, B***).We observed a significant increase in the miR-130a level but no significant change in precursor miR-130a in primary HPASMCs treated with H+C EVs when compared with cells treated with EVs derived from only HIV-1-infected, only cocaine-treated, or untreated MDMs. To further validate the direct role of miR-130a in modulating PTEN expression, HPASMCs were transfected with either antagomir 130a (AmiR-130a) or with scrambled antagomir (AmiR-scr) followed by treatment with or without H+C EVs. As expected, decrease in the expression of PTEN mRNA in HPASMCs upon addition of H+C EVs was rescued in the presence of Amir-130a as compared to the untransfected and AmiR-scr controls (Fig. 5*C*). Further silencing miR-130a with its antagomir also prevented the decrease in the expression of TSC-1 and -2 mRNA in HPASMCs treated with H+C EVs (Fig. 5*D, E*). This finding dovetailed with decrease in H+C EV-mediated HPASMCs proliferation when the effect of miR-130a was inhibited by transfection of cells with antagomir 130a (Fig. 5*F*). This clearly suggests that transfer of miR-130a from EVs derived from H+C-treated macrophages play a crucial role in stimulating smooth muscle proliferation by directly downregulating the PTEN expression which, in turn, activates downstream AKT signaling.

## DISCUSSION

Increased survival of HIV-infected patients with the advent of antiretroviral therapy has resulted in an increase in the prevalence of noninfectious complications including pulmonary arterial hypertension (42–44). Furthermore, illicit drug use has been identified as one of the most common risk factors in the individuals with HIV-associated PAH (9, 10, 43). We demonstrated enhanced pulmonary vascular remodeling in HIV-infected lung tissues from intravenous cocaine and opioid abusers. In addition, we demonstrated that cocaine potentiates the effect of HIV proteins in promoting proliferation of pulmonary SMCs leading to increased mean pulmonary arterial pressure and right ventricular systolic pressure in noninfectious HIV-transgenic rats. Therefore, proteins released by HIV-infected cells such as macrophages and T cells play a pivotal role in the dysfunction of uninfectable vascular cells. In the current study, we provided evidence of EVs as another way of communication between HIV-infected cells and vascular cells that may be involved in the development of pulmonary arteriopathy.

Inflammation plays a crucial role in vascular remodeling associated with chronic lung, neurologic and cardiovascular diseases associated with HIV infection (5, 45–47). Various reports demonstrate the presence of inflammatory cells within the plexiform lesions and in the vicinity of pulmonary vessels with intimal and medial wall thickening in pulmonary hypertension patients (48–50). Morris *et al.* (5) suggested the presence of chronic obstructive pulmonary disorder and pulmonary hypertension as secondary effects of chronic inflammation in patients with HIV. Furthermore, elevated levels of inflammatory markers were found in patients infected with HIV-1 through IVDU compared with other routes of infection (51). We observed significant perivascular inflammation in the lung sections from HIV-infected humans (9) and SIV-infected macaques exposed to illicit drugs (10) including an increased number of macrophages in the remodeled thickened vessels. Consistent with these findings, we also reported an infiltration of mononuclear cells near or within the adventitia of the thickened vessels in the HIV-Tg rat model in the absence or presence of cocaine (12, 18). We observed significant pulmonary artery medial hypertrophy with right ventricular hypertrophy in Sprague Dawley (SD) strain of HIV-Tg rats in the absence of cocaine (18). However, in Fischer HIV-Tg rats, we observed an elevated mean pulmonary arterial pressure and right ventricular systolic pressure, as well as increased SMC proliferation in the HIV-Tg rats only after exposure to cocaine in both 4- and 9-mo-old rats. Strain specific differences in pulmonary vascular remodeling have been reported earlier (52) and when we compared 2 strains, we observed severe perivascular inflammation in SD HIV-Tg rats (compared with that in Fischer HIV-Tg rats), that corresponded with significantly more vascular remodeling in SD rats. Also these inflammatory cells were abundantly positive for HIV proteins, such as Tat or negative regulatory factor, whereas relatively very few SMCs were found to be positive for viral proteins within the remodeled vessels. These findings suggest the importance of crosstalk between inflammatory and SMCs during pulmonary arteriopathy.

Given that vascular cells do not support productive viral replication, our current findings suggest that, in addition to viral proteins, EVs released by the surrounding HIV-infected macrophages and T cells act as mediators during the synergistic contributions of HIV-1 and cocaine to pulmonary vascular remodeling. We found that the addition of EVs released from H+C-treated MDMs to pulmonary SMCs results in enhanced proliferation of cells and this increase was significantly more compared to the effect of EVs from only HIV-infected or only cocaine-treated macrophages. This finding corresponded to the increase in the number of EVs released by macrophages on combined treatment compared to monotreatment. The marginal increase in the proliferation of SMCs on exposure to EVs from monotreatment group macrophages was in accordance with our previous *in vitro* as well as *in vivo* published findings where we demonstrated that a “dual hit” of HIV and cocaine significantly exacerbates smooth muscle hyperplasia and PAH phenotype compared to a “single hit” of either cocaine or viral proteins (9, 12). In recent years, the role of EVs in intercellular communication has been well established (53) in the pathogenesis of various diseases, including pulmonary hypertension (54, 55). Plasma exosomes from mice with monocrotaline-induced PAH were demonstrated to cause pulmonary hypertension in healthy mice and this effect was reversed by administering exosomes derived from mesenchymal stem cells (23). Mesenchymal stem cell–derived exosomes have also been reported to help suppress the hypoxia-induced lung inflammation, thereby mitigating vascular remodeling and development of hypoxic pulmonary hypertension (22, 56). Furthermore, the role of HIV infection in formation and secretion of EVs is well known (57, 58). It has been reported that HIV-1 modulates the secretion and packaging of EVs to promote its infection (43, 57, 59, 60). Also, HIV-1 virions can get packaged within the EVs to escape immune surveillance and replicate. Viral proteins, such as negative regulatory factor have been found in HIV-infected cell–derived EVs that have the ability to cause immune activation as well as promote neuroimmune or cardiovascular complications (43, 46, 61, 62). Cocaine is known to impair the functions of macrophages and CD4^+^ lymphocytes. We earlier demonstrated an augmented expression of activation marker major histocompatibility complex class II and increase in viral replication in HIV-1–infected macrophages on exposure to cocaine (24). In this study, we report that cocaine also increases the production and release of EVs by these infected macrophages.

It has been suggested that different cell types can release or receive miRNAs *via* EVs as the mode of communication to initiate various cellular process including proliferation associated with PAH (23, 63, 64). Recently, it has been reported that immune-activated monocyte–derived exosomes change expression of miRNAs in brain endothelial cells to trigger inflammation and cause brain vascular dysfunction (62). Moreover, there is a substantial amount of evidence suggesting involvement of miRNAs in modulation of SMCs proliferation and vascular remodeling in cardiovascular diseases and pathogenesis of PAH (65–67). However, for the first time, we report alterations in the miRNA cargo packaged in EVs released by HIV-infected and cocaine-treated human MDMs. Small-RNA seq analysis revealed PI3K/AKT signaling as one of the major pathways targeted by differentially regulated miRNAs (66) in EVs derived from H+C macrophages. The role of PI3K/AKT signaling in development of PAH has been described. It has been reported that inhibition of the PI3K/AKT signaling leads to suppression of hypoxia-induced pulmonary vascular remodeling by attenuating smooth muscle proliferation (68–70). Furthermore, many studies have shown direct involvement of PTEN inhibition in the potentiation of vascular smooth muscle proliferation (71–73). We earlier reported that addition of cocaine and the HIV protein Tat leads to platelet-derived growth factor receptor-β–induced activation of downstream PI3K/AKT signaling, resulting in smooth muscle hyperplasia (74). In this study we demonstrate that exposure of SMCs to the altered miRNA cargo of EVs released by H+C-treated macrophages promotes smooth muscle hyperplasia by inhibiting the expression of PTEN and TSC1/2 complex, both negative regulators of PI3K/AKT signaling (75). We found significant upregulation of miR-130a and -27a in EVs derived from H+C-treated macrophages compared to EVs from untreated cells; however, the level of miR-130a in H+C EVs was also significantly upregulated when compared to EVs derived from macrophages treated with only HIV-1 or cocaine.

The role of miR-130a in promoting cell proliferation and tumorigenesis has been well studied. Recently, Brock *et al.*, (76) reported a role of miR-130a in hypoxia-induced smooth muscle proliferation leading to pulmonary hypertension. Specifically, miR-130a is known to negatively regulate PTEN expression in various types of cancer (77) by directly binding to the 3′UTR of PTEN (37–40). Because we observed no significant change in the expression of endogenous precursor miR-130a level upon addition of H+C EVs over HPASMCs, our findings suggest transfer of mature miR-130a from EVs to HPASMCs that leads to inhibition of PTEN and therefore activation of PI3K/AKT-dependent smooth muscle proliferation. This is supported by recent *in vivo* study that reports brain metastasis on transfer of PTEN targeting miRNAs from astrocytes to tumor cells *via* astrocyte-derived exosomes (78). Furthermore, Wang *et al.* (41) recently reported miR-130a to directly target TSC-1/2 also and activate *m*-TOR pathway. Likewise, we observed decreased mRNA expression of TSC-1/2 on exposure of HPASMCs to H+C EVs, and this decrease was rescued in HPASMCs transfected with antagomir-130a.

**Figure 6:**
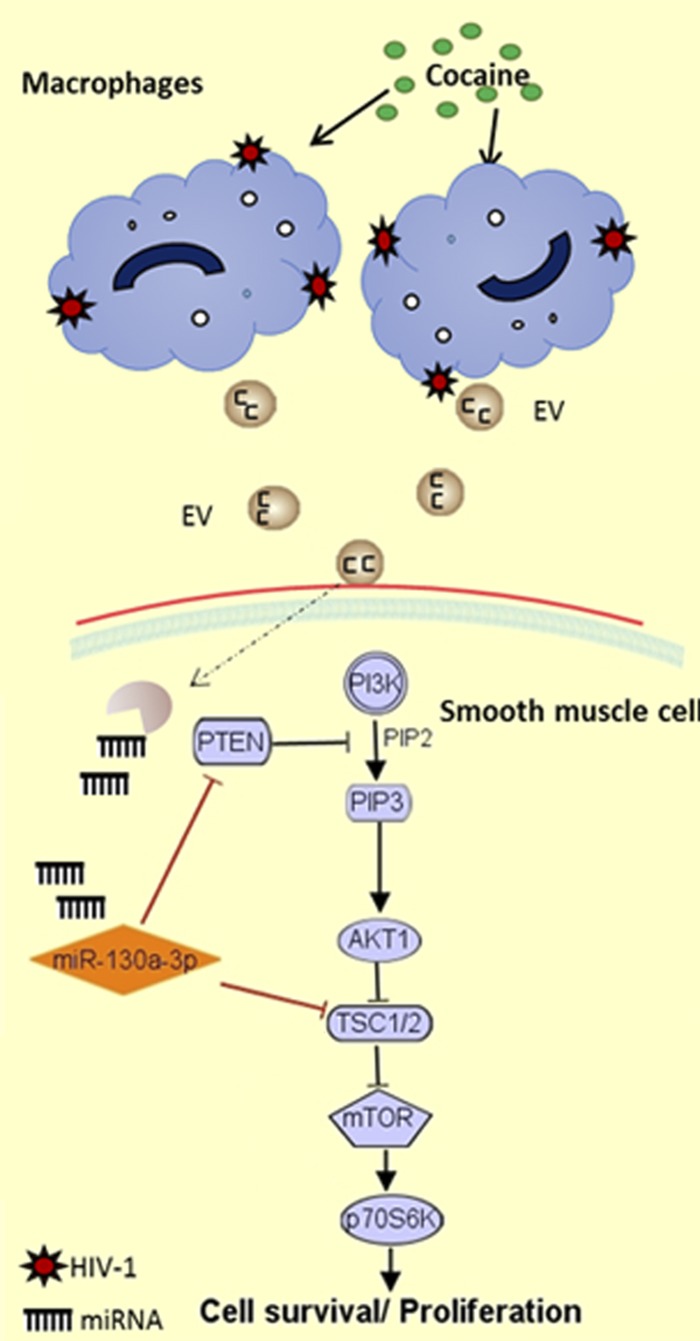
Schematic showing miR-130a-mediated regulation of PI3K/AKT signaling on exposure to HIV-infected, cocaine-treated, and macrophage-derived EVs.

In summary, results support the contribution of inflammatory cell–derived EVs to the pathogenesis of PAH by delivery of specific miRNAs. We demonstrated increase in the release of EVs and change in EV miRNA cargo by HIV-1–infected, cocaine-exposed macrophages compared to monotreatments. These H+C-treated macrophage–derived EVs were found to promote hyperproliferation of pulmonary arterial SMCs by delivery of proproliferative EV miRNA cargo. Specifically, we demonstrate the involvement of EV miR-130a in EV mediate downmodulation of PTEN expression and activation of PI3K/AKT signaling, leading to SMC proliferation (**Fig. 6**). Nevertheless, we also expect EVs to carry many other proliferative miRNAs, as observed in small-RNA seq analysis, along with proteins and transcription factors (19, 79) such as HIF-1α, cytokines (80), and growth factors that may contribute to enhanced proliferation of cells. Overall, our study underscores the role of communication between HIV-1–infected inflammatory cell–derived EVs and uninfectable pulmonary vascular cells in HIV PAH pathogenesis.

## Supplementary Material

This article includes supplemental data. Please visit *http://www.fasebj.org* to obtain this information.

## Abbreviations

AKT: protein kinase B, EV, extracellular vesicle
FBS: fetal bovine serum
H+C: HIV+cocaine
HPASMC: human pulmonary arterial smooth muscle cell
HRPAH: HIV-related pulmonary arterial hypertension
IPA: Ingenuity Pathway Analysis
IVDU: intravenous drug use
MDM: monocyte-derived macrophage
PAH: pulmonary arterial hypertension
PCNA: proliferating cell nuclear antigen
PTEN: phosphatase and tensin homolog
RNA seq: RNA sequencing
SD: Sprague-Dawley
SMC: smooth muscle cell
Tat: transactivator of transcription
TEM: transmission electron microscopy
Tg: transgenic
TSC: tuberous sclerosis
