# Supplementary material for: Macrophage-derived extracellular vesicles mediate smooth muscle hyperplasia: role of altered miRNA cargo in response to HIV infection and substance abuse
Source: FASEB J. 2018 Apr 19;32(9):5174–85. doi: 10.1096/fj.201701558R (PMC6103174; doi:10.1096/fj.201701558R)
Supplement: Supplementary file 1 [file fj.201701558R.sd1.docx]

**Macrophage derived Extracellular Vesicles mediated Smooth Muscle Hyperplasia: Role of altered miRNA cargo in response to HIV-infection and substance abuse**

Himanshu Sharma^1^, Mahendran Chinnappan^1^, Stuti Agarwal^1^, Pranjali Dalvi^1^, Sumedha Gunewardena^2^, Amy O`Brien Ladner^1^ Navneet K. Dhillon^1,2^

^1^Division of Pulmonary and Critical Care Medicine, Department of Medicine, University of Kansas Medical Center, Kansas City, KS-66160, USA.

^2^Department of Molecular & Integrative Physiology, University of Kansas Medical Center, Kansas City, KS-66160, USA.

**SUPPLEMENTAL MATERIAL**


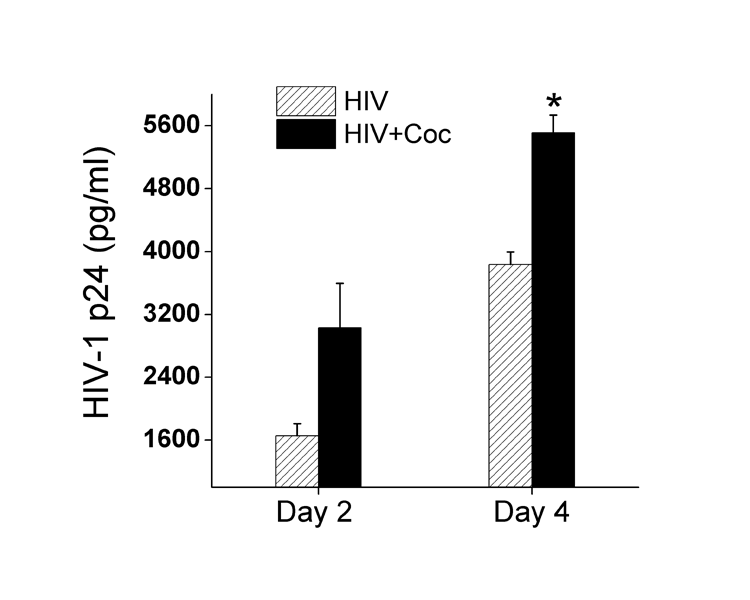


**Figure S1:** Increased viral load in supernatants from HIV infected and cocaine treated macrophages as determined by p-24 ELISA. *p<0.05 compared to HIV.


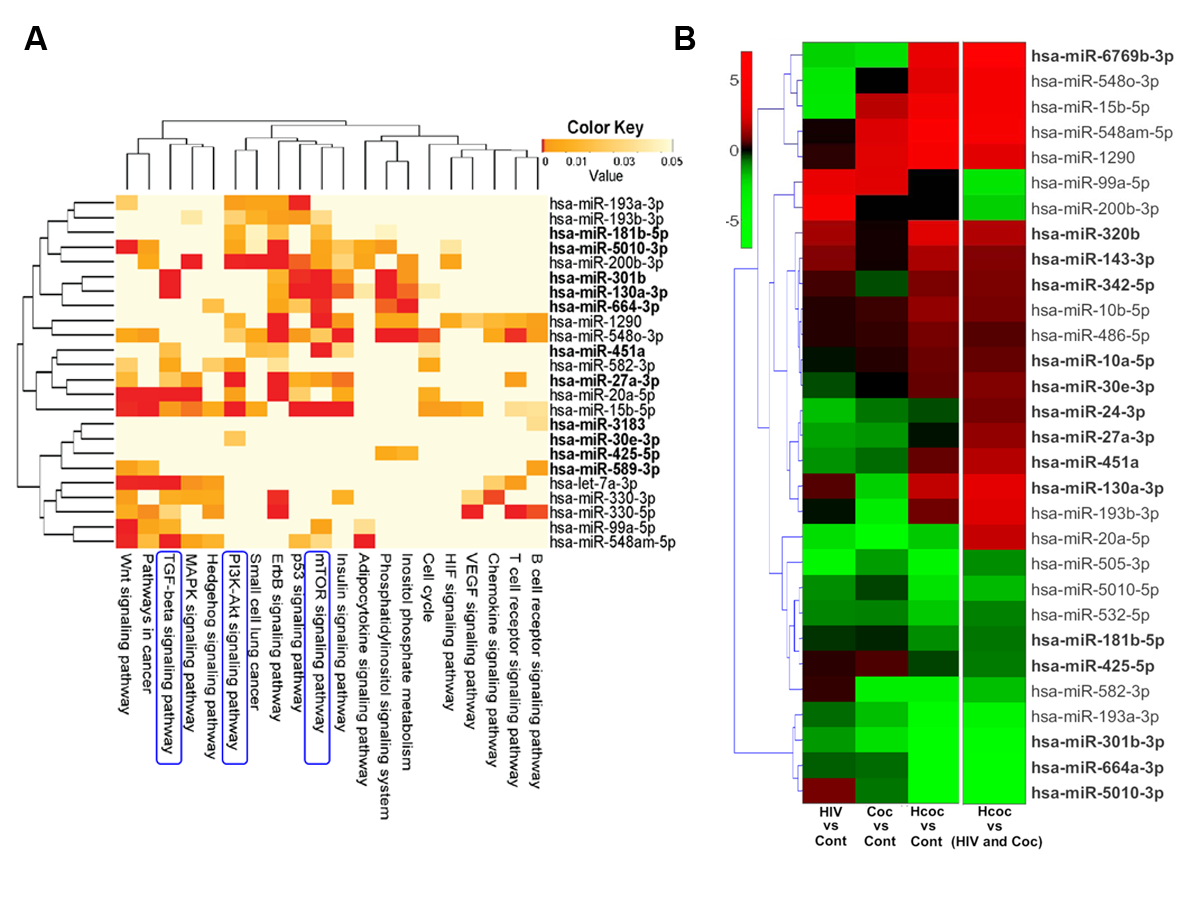


**Figure S2: (A)** Schematic representation created with DIANA mirPath v3.3 software showing upregulated miRNAs with predicted target pathways. **(B)** The color gradient in Heat map reflects the log fold changes in PI3K/AKT signaling related EV miRNAs. miRNA with p<0.05 are shown in bold.
